# Six-month evaluation of normal mouse brain side effects: Comparing FLASH and conventional proton partial brain irradiation

**DOI:** 10.1016/j.ctro.2026.101183

**Published:** 2026-05-09

**Authors:** Manuel Bernabei, Sindi Nexhipi, Elisabeth Bodenstein, Felix Horst, Armin Lühr, Jörg Pawelke, Moritz Schneider, Michael Schürer, Roman Schwarz, Antje Dietrich, Elke Beyreuther

**Affiliations:** a OncoRay – National Center for Radiation Research in Oncology, Faculty of Medicine and University Hospital Carl Gustav Carus, TUD Dresden University of Technology, Helmholtz-Zentrum Dresden-Rossendorf, Dresden, Germany; b Helmholtz-Zentrum Dresden-Rossendorf, Institute of Radiation Physics, Dresden, Germany; c Helmholtz-Zentrum Dresden-Rossendorf, Institute of Radiooncology – OncoRay, Dresden, Germany; d German Cancer Consortium (DKTK), Partner Site Dresden, and German Cancer Research Center (DKFZ), Heidelberg, Germany; e Department of Physics, TU Dortmund University, Dortmund, Germany; f Department of Radiotherapy and Radiation Oncology, Faculty of Medicine and University Hospital Carl Gustav Carus, TUD Dresden University of Technology, Dresden, Germany; g National Center for Tumor Diseases (NCT), NCT/UCC Dresden, a partnership between DKFZ, Faculty of Medicine and University Hospital Carl Gustav Carus, TUD Dresden University of Technology, and Helmholtz-Zentrum Dresden-Rossendorf (HZDR), Germany

**Keywords:** Brain FLASH radiotherapy, Long-term side effects, Microglia density

## Abstract

**Background and purpose:**

Preclinical studies demonstrated ultra-high dose-rate radiation treatment ability to effectively control local tumour growth while reducing damage to healthy tissues. This methodological pilot study was designed and conducted to establish an irradiation setup and to generate data for future studies investigating long-term side effects of mouse brain irradiation with proton FLASH (FLASH-PT) and conventional proton therapy (CONV-PT).

**Material and methods:**

Fourteen C57BL/6JRj female mice were partial brain irradiated with a single dose of 22.5 Gy using a proton spread-out Bragg peak (SOBP). Mice were randomised into FLASH-PT (268 Gy/s, n = 6), CONV-PT (0.27 Gy/s, n = 6), and unirradiated sham groups (n = 2). In addition to skin reaction monitoring as short-term endpoint, regular contrast-enhanced magnetic resonance imaging (MRI) was performed during the six-month follow-up. Brains were formalin-fixed and paraffin-embedded and stained to assess activation of microglia.

**Results:**

The analysis of skin reactions demonstrates a trend in tissue-sparing effect for the FLASH group. In the brain MRI, no contrast enhancement was observed after irradiation. Although no group–level differences in microglia activation were observed between FLASH-PT and CONV-PT, both treatments showed increased density of activated microglia, which was distinct in specific regions within the periventricular area.

**Conclusion:**

The established irradiation setup allowed for a clinically relevant approach to preclinical FLASH-PT research. Partially irradiated brain showed no microglia-related FLASH effect. Further studies are required to fully comprehend dose–effect curves and timing for the FLASH effect in the brain.

## Highlights

• Clinically relevant brain sub-volume irradiation is feasible with SOBP at UHDR.
• FLASH-PT delayed acute skin toxicity compared to CONV-PT.
• No MRI-detectable radiation-induced brain lesions were observed up to 6 months after 22.5 Gy of either FLASH-PT or CONV-PT.
• FLASH-PT and CONV-PT induce the same density of microglia activation six-months post-irradiation.
• This pilot study defines feasibility and variability, guiding future mechanistic and dose–effect FLASH studies.

## Introduction

Proton radiotherapy (PT) is considered as one of the leading treatment options for both paediatric and adult brain tumour patients [1]. The characteristic dose distribution of protons with the dose maximum (Bragg peak) at the end of their range has enhanced healthy tissue sparing capacity compared to traditional photon-based delivery techniques [2]. Improved treatment outcomes result in longer survival, thereby increasing attention to late side effects of brain radiotherapy [3].

In the clinical context, patients may develop radiation-induced brain injury (RIBI), a potential complication often detected as contrast enhancement with magnetic resonance imaging (MRI) [4]. This radiological finding may precede neurological symptoms, as brain irradiation has been associated with a risk of long-term neurocognitive decline [5], [6]. In this context, research on new delivery techniques for proton therapy could further reduce long-term side effects and improve survival [7].

Ultra-high dose-rate (UHDR) radiotherapy has the potential to widen the therapeutic window by reducing the noxious effect of radiotherapy towards healthy tissues while maintaining comparable tumour control probability relative to conventional radiotherapy, a phenomenon known as the “FLASH effect” [8], [9]. Delivering radiation at UHDR (> 40 Gy/s) can induce the FLASH effect; however, the magnitude of this effect may vary depending on the study endpoint and the parameters of the delivered radiation dose [10], [11]. Most preclinical studies investigating the FLASH effect in the brain used whole–brain irradiation [12], [13], [14]. In contrast, partial brain irradiation, common in clinical practice, may lead to distinct molecular, cellular and neurocognitive effects [15] that consequently impact the FLASH effect.

To elucidate the long-term effects of PT, Suckert et al. [16] have developed a preclinical mouse model of RIBI to closely mimic the focal irradiation strategies commonly employed in clinical practice. After proton irradiation, preclinical MRI scans exhibited contrast-enhancing lesions (CEL) indicating blood–brain-barrier breakdown and mirroring the clinical situation of RIBI. Histological analyses of these irradiated mouse brains showed increased density and activation of microglia [17] and astrocytes [18] in irradiated areas. Activated microglia drive and amplify the neuroinflammatory response to the radiation insult [19]. Unresolved sustained microglial activation and astrogliosis create a chronic neuroinflammatory state that impairs neurogenesis, leading to cognitive deficits [20].

Adapting the mouse model of Suckert et al. [16] to investigate RIBI sequelae, this pilot study aimed at evaluating the long-term healthy tissue complications induced by UHDR proton treatment (FLASH-PT) in comparison to conventional proton treatment (CONV-PT). The necessary ultra-high dose-rates were produced in a spread-out Bragg peak (SOBP) proton beam, which was directed through a sub-volume of the brain encompassing both hippocampi. Afterwards, to determine a potential long-term FLASH sparing effect, regular MRI scans were performed over a six-month follow-up period to monitor the development of brain lesions, and the microglial response to radiation was evaluated through subsequent histological analysis. During the follow up, mice were scored for skin reaction to have an independent validation of a FLASH effect in an acute reacting tissue.

## Material and methods

### Partial brain irradiation

**Fig. 1:**
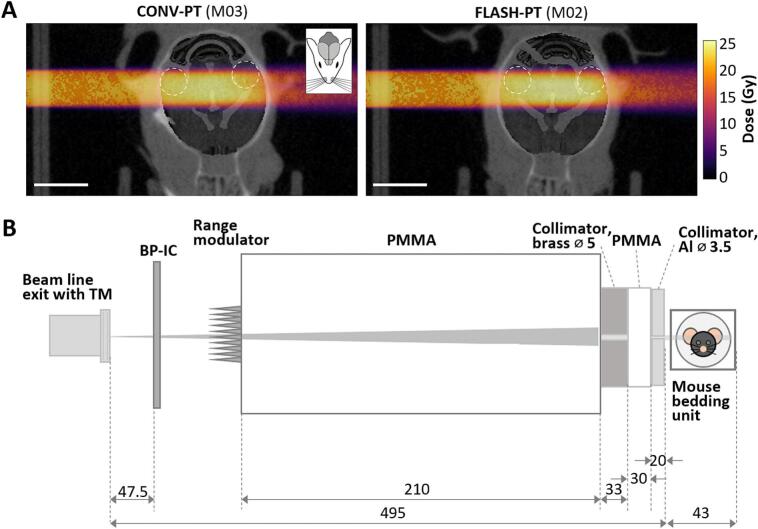
CONV-PT and FLASH-PT dose distributions with schematic of the irradiation setup. (A) Simulated proton dose distributions for two irradiated mice. The beam path, directed from left to right, encompasses the whole length of the mouse head in both CONV-PT (left) and FLASH-PT (right) treatments. Dose distributions are overlaid onto a transversal brain slice at a depth corresponding to the beam centre, which is registered with the DSURQE mouse brain atlas [41]. The location of the hippocampi is indicated by white dashed circles. A mouse icon is included to give anatomical references about the orientation of the mouse head. Scale bar = 5 mm. (B) Schematic representation of the irradiation setup comprising the individual components and respective distances. From left to right: beam line exit with transmission ionization chamber (TM), Bragg peak ionization chamber (BP-IC) as a second independent beam monitor, 3D-printed range modulator, Polymethylmethacrylat (PMMA) range shifter, brass collimator with 5 mm diameter opening, PMMA, aluminium collimator with 3.5 mm diameter opening, mouse bedding unit enclosing the anaesthetised mouse. All measures are in mm.

This animal experiment was conducted in accordance with Directive 2010/63/EU, the German Animal Welfare Act (TierSchG), the Animal Welfare Experimental Animals Ordnance (TierSchVersV), and with the approval of the Landesdirektion Sachsen (DD24.15131/449/32). Fourteen 10-week-old C57BL/6JRj female mice were randomised into three experimental groups: FLASH-PT (n = 6), CONV-PT (n = 6), and sham (n = 2). Irradiation was performed at the horizontal fixed-beam beamline in the experimental hall of OncoRay, Dresden [21], [22]; a comprehensive summary on beam and dose delivery parameters according to Böhlen et al. [23] can be found in Table 1. Briefly, a 225 MeV proton beam was modulated with a 3D-printed range modulator (VisiJet M2S-HT250 printing material) [24] to obtain a SOBP of 1.5 cm width penetrating the full medial length of the mouse head (Fig. 1A). The proton beam was laterally collimated to achieve an irradiation field of 5 mm in diameter targeting only a sub-volume of the brain. Each mouse received either 0 or 22.5 Gy in a single fraction. FLASH-PT was delivered at UHDR with 268 Gy/s at the centre of the beam and above 100 Gy/s in the major part of the irradiation field. CONV-PT was delivered at 0.27 Gy/s at the centre of the beam. The doses delivered were 22.6 ± 0.11 Gy and 22.5 ± 0.02 Gy for FLASH-PT and CONV-PT, respectively, with a skin entrance dose of 23.9 Gy and skin exit dose of 21.2 Gy. For the irradiation, mice were anaesthetised following the same protocol described in Suckert et al. [16]. Preclinical studies [25], [26] have shown that the neuroprotective FLASH effect depends on an oxygen threshold and disappears when animals are anaesthetised with isoflurane in an oxygen mixture. To avoid any influence of inhaled oxygen during irradiation, mice were anaesthetised with ketamine (100 mg/kg) and xylazine (10 mg/kg), positioned in a temperature-controlled bedding unit, and fixed on a motorized stage. Correct alignment of the hippocampus within the central axis of the proton beam was achieved by using proton radiography and dedicated in-house software [27]. A schematic representation of the entire irradiation setup is provided in Fig. 1B. A detailed description of the irradiation setup, SOBP formation and field shaping, mouse positioning including proton radiography, dosimetry as well as accompanying proton transport simulations are provided in the Supplementary material.

**Table 1:** Parameters used for irradiating the mouse brains with UHDR and conventional proton beams.

Minimal Reporting	UHDR	CONV
**General description:**		
Device name:	ProteusPLUS proton therapy system (IBA, Louvain-la-Nueve, Belgium)	
Accelerator type:	230 MeV isochronous cyclotron (IBA C230)	
Dose delivery technique:	Static pencil beam, horizontal	
Traceability and dosimetry code of practice used:	TRS-398; ionization chamber (IC) calibrated for absorbed dose to water using ^60^Co photons at PTW (IC manufacturer) traceable to primary standard at PTB (German metrology institute); microdiamond calibrated against IC	
Additional key information about delivery:	Dose delivery controlled via in-house developed beam control system; beam monitoring via a beam line integrated monitor chamber and a Bragg Peak IC	
	Dose delivery controlled over pulse length, set by cross-calibration of Bragg Peak IC (charge read-out) to microdiamond at sample position considering 5 % saturation effect of the IC	Dose delivery controlled over monitor units of the build-in monitor chamber calibrated against microdiamond at sample position
Preclinical: Biological system(s), model(s), endpoint(s):	Female C57BL/6JRj, endpoint: skin toxicity and microglia activation	
Additional key information (including imaging) about irradiated systems/models/patients	Imaging: proton radiography of each mouse directly before treatment	

**Non-temporal beam parameters (per beam):**		
Radiation type and nominal beam energy:	Protons, modulated 225 MeV SOBP	
Cyclotron current [nA]:	488	0.48
Beam current at target site [nA]:	215	0.21
Beam dose at reference point [Gy]:	22.5	
Reference point:	Entrance in mouse head, measurement at head position with microdiamond	
Source-to-surface distance:	Approx. 50 cm taking the beam exit window as source (see Fig. S1)	
Field size:	See Fig. S2: maximum field size laterally ∼ 5 mm, maximum dose within 3.5 mm	
Treated volume:	5 mm laterally times thickness of mouse head of approx. 14 mm	

**Temporal beam structure parameters (per beam):**		
Beam delivery mode:	Quasi-continuous beam (bunch repetition frequency of 106 MHz)	
Beam-on-time [s]:	0.083	85
Time to start of next beam:	n.a. (single beam/field and single dose fraction, respectively)	
Number of pulses for beam:	1 pulse	Quasi-continuous beam
Pulse length [ms]:	83	n.a.
Pulse repetition frequency:	n.a. (single pulse)	
Pulse charge [nC]:	17.8	n.a.

**Derived and additional parameters:**		
Average dose rate at reference point [Gy/s]:	268	0.27
Dose per pulse [Gy]:	22.5	
Representative 2D dose distribution of beam, PDD and lateral profiles:	Lateral profiles, see Fig. S2

### Follow-up

Following irradiation, mice were monitored twice a week for 180 days, including scoring of radiation-induced skin reaction following the RTOG/EORTC guidelines [28]. Serial contrast-enhanced MRI scans were performed at defined time points: 3, 6, 9, 12, 16, 20, and 24 weeks post-irradiation, with intraperitoneal injections of Gadolinium-based contrast agent (Magnevist (0,5 mmol/ml, 100 µl), Bayer, Leverkusen, Germany). For the procedure, anaesthesia was induced via 1–2 % isoflurane inhalation mixed with oxygen (Baxter Deutschland GmbH Medication Delivery, Unterschleißheim, Germany). T1-weighted MRI images were acquired with BioSpec 3 T Preclinical MRI System (Bruker, Billerica, MA, USA) to detect CELs. Because of technical reasons, MRI data prior to week 12 were unavailable for analysis.

### Histology

At the end of the follow-up, the mice were sacrificed, and the brains were formalin-fixed and paraffin-embedded (FFPE), as previously described [16], [29]. Brains were sliced into transverse sections of 3 μm each at 100 μm intervals at the depth corresponding to the irradiated volume, yielding 5–6 histological slides for evaluation [17]. Ionized calcium-binding adaptor molecule 1 (Iba1, NB100-1028, Novus Biologicals, Centennial, CO, USA) was used to label microglia. Iba1 regulates cytoskeletal dynamics and remodelling, making it a useful marker for studying microglial morphology. All slides were counterstained with DAPI (4′,6-diamidino-2-phenylin-dole, ab228549, Abcam, Cambridge, UK) for nuclei detection. Whole slide imaging was performed using the AxioScan.Z1 slide scanner microscope (Carl Zeiss, Germany) of the Light Microscopy Facility, a core facility of the CMCB Technology Platform at TU Dresden. Stained slices were imaged with a 10x objective. The staining protocol is provided in the Supplementary material.

### Image analysis

**Fig. 2:**
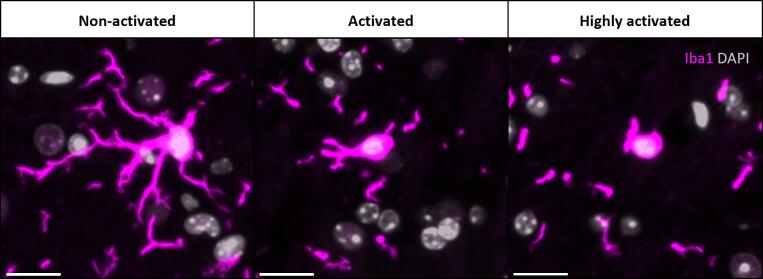
Morphological arrangement of microglia in different activation statuses. The figure shows representative microglia cells in different activation statuses. Non activated with ramified morphology (M Score > 0 and ≤ 1), activated with an ameboid shape (M Score > 1 and ≤ 2), and highly activated with a rounded morphology (M Score > 2). Scale bar = 20 µm.

All whole–brain images were analysed using the image analysis software FIJI (ImageJ version 2.9.0, National Institute of Health, USA) [30]. Microglia analysis was performed by applying an algorithm developed by Nexhipi et al. [17]. This algorithm segmented the microglia cells and based on the morphology of the cells, divided them into three groups of activation: non-activated, activated, and highly activated. The degree of activation was based on cells’ circularity and area to calculate the M Score [31]. Activated microglia cells tend to rearrange their cytoplasmic configuration, reducing podia branching by adopting a more compact rounded shape. These morphological changes yield higher circularity values [32], and consequently higher M Scores, indicating increased activation status [33] (Fig. 2). Analysis was performed over the whole–brain area, excluding the olfactory bulb, *cerebellum*, *pons*, and *medulla*.

### Statistical analysis

The Mantel–Cox test was used to determine the significance of the difference in development time of radiation-induced skin reaction between FLASH-PT and CONV-PT. Welch́s *t*-test (two-tailed) was used to compare the different microglia densities between FLASH-PT and CONV-PT. Statistical significance was defined as not significant (p ≥ 0.05), significant (p < 0.05) with increasing levels of significance (p < 0.01, p < 0.001) reflecting stronger evidence against the null hypothesis.

## Results

### Acute skin reaction is delayed for FLASH-PT vs. CONV-PT

**Fig. 3:**
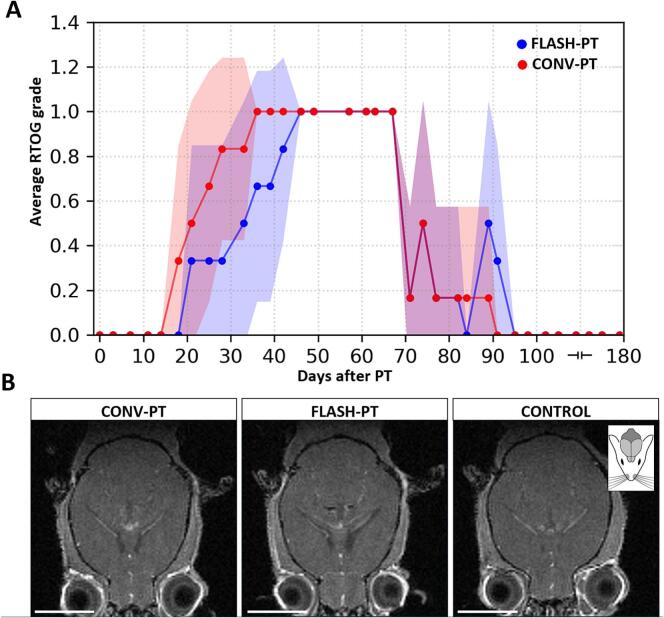
Timeline of the development of radiation-induced skin reactions after proton treatment and end-of-follow-up MRI scans. (A) The figure shows the development and recovery of the skin reactions for the FLASH-PT and CONV-PT groups. Data points represent the average of RTOG values per mouse in treatment group obtained on scoring day. Shading represents the standard deviation of the average value. (B) Representative images of the T1-weighted MRI scans at 24 weeks post-irradiation of selected mice for each treatment group: CONV-PT (left), FLASH-PT (middle) and sham (right). A mouse icon is included to give anatomical references about the orientation of the mouse head. Scale bar = 5 mm.

The analysis of the skin reaction was performed for erythema and hair loss, which were the only side effects visible in the mice (RTOG grade 1). A distinct difference of the temporal development of acute skin reactions induced by radiation treatment was observed between the CONV-PT and FLASH-PT mice groups. By the fifth week after irradiation, all the CONV-PT treated mice exhibited skin reactions at the irradiated area. In contrast, the totality of the FLASH-PT treated mice presented skin reactions by the middle of the seventh week, showing a clear delay compared to the CONV-PT treatment. From the tenth week onward, the mice in both groups started to gradually recover from the skin-related complications. Indeed, mice with hair loss started to grow back white fur in the irradiated area of the head. Mantel-Cox test (χ^2^ = 2.89, df = 1, p = 0.089) confirms a trend towards delayed development of skin reactions for the FLASH-PT mice group (Fig. 3A).

### No contrast-enhancing lesions were detected over the length of the follow-up

None of the T1-weighted MRI images of the six-month follow-up period showed evidence of contrast enhancement in any irradiated mice as well as on control mice. Representative images at 24 weeks post-irradiation at a brain depth corresponding to the depth of the histological slides are shown in Fig. 3B.

### Density of activated microglia increases in the periventricular region

**Fig. 4:**
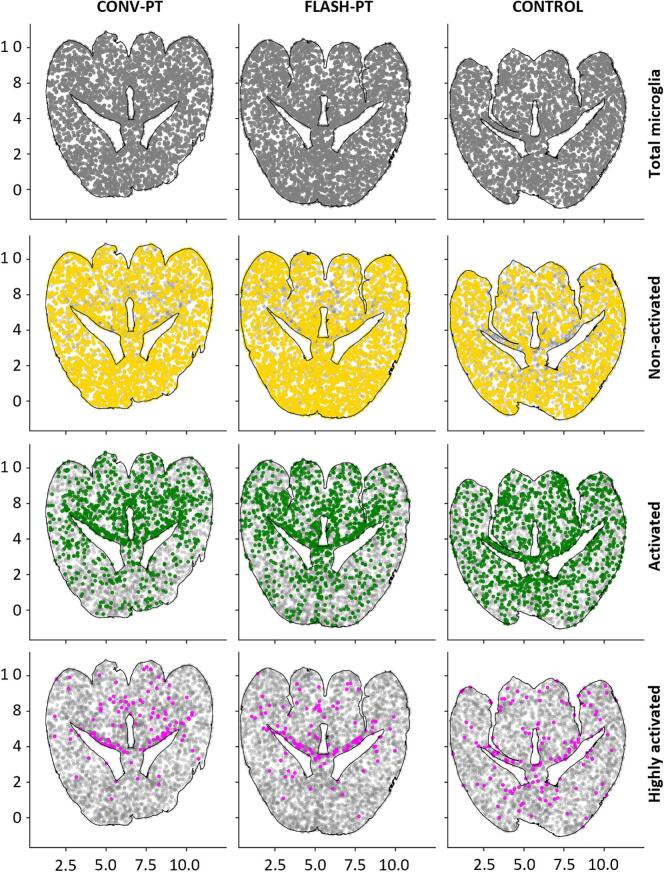
Microglia density distribution maps over the whole–brain area. The figure represents the microglia distribution for selected mice in different treatment groups at matched beam centre positions. The images were created by plotting the centre-of-mass coordinate point of each microglia in the slice and then color-coded based on the activation status. Total microglia population is reported as reference in grey colour in all the maps. Scale system is in mm.

**Fig. 5:**
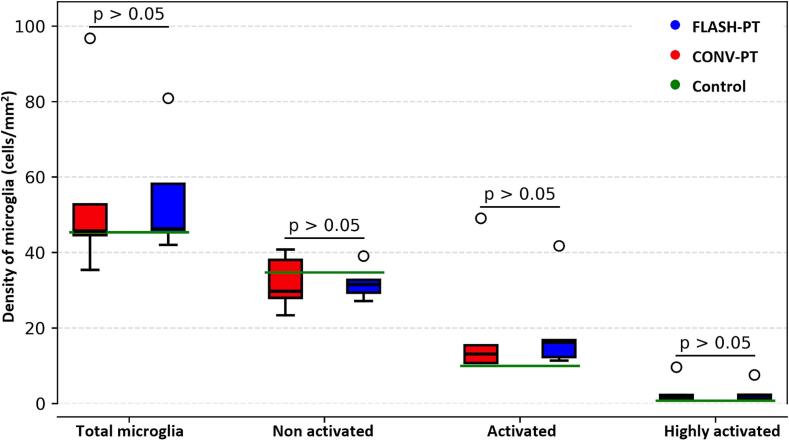
Microglia density for the different activation statuses over the whole–brain area. The figure shows box plots of microglia density for different activation states across the brain. For each mouse, 5–6 brain slices were analysed and the densities averaged to obtain a single value per mouse. These single values were then used to calculate group averages for the FLASH-PT and CONV-PT groups. Boxes indicate the interquartile range with black lines indicating the median value for the group. Dots represent single mouse values with data points beyond the whiskers considered as outliers. Green lines represent the control mouse value (average of 5 brain slices, only 1 control mouse). (For interpretation of the references to colour in this figure legend, the reader is referred to the web version of this article.)

Five brains per treatment group and one control brain were considered for sectioning and subsequent analysis. Fig. 4 presents the microglia density across the whole–brain area for the three different states of microglia activation in representative mice. Within the same irradiation group, inter-mouse differences in the spatial distribution of microglia were observed (Supplementary material Fig. S4.1 and S4.2). In general, the density of non-activated microglia appears uniform across the whole–brain area for both irradiated groups. As activation state increases, areas of high microglia activation density progressively localise to the lateral periventricular regions. Interestingly, this increase was concentrated specifically at the site closer to the midbrain that lies over the fibre tract. No statistically significant difference was found between the FLASH-PT and CONV-PT groups for the density of non-activated, activated and highly activated microglia across the whole–brain area (Fig. 5). A table of brain slices analysed per mouse (Table S1) and representative activation maps (Fig. S4.1 and S4.2) are reported in the Supplementary material.

## Discussion

In this pilot study we evaluated the long-term brain tissue complications after FLASH-PT in comparison to CONV-PT. While most preclinical studies involved whole brain irradiation, the partial brain SOBP dose delivery represents the clinical situation for tumour treatment. In this situation, the FLASH effect could help reducing potential side effects to normal tissue. Regular MRI scans over the six-month follow-up period were performed to oversee any contrast-enhancement that may be caused by the treatment. At the end of the follow-up, we examined microglia activation finding no differences between FLASH-PT and CONV-PT irradiation. However, a trend towards a delayed onset of skin reaction for FLASH-PT was observed. This pilot study evaluated the feasibility of the described irradiation setup and generated preliminary data to guide future comparisons between FLASH-PT and CONV-PT treatments. This type of studies implies low statistical power deriving from the small sample size. Indeed, the aim was estimating group variability and effect size to plan more advanced confirmatory experiments.

Many preclinical studies have shown a protective FLASH-PT effect in early responding tissues, such as skin [34], [35]. In our study, the onset of radiation-induced erythema and hair loss tended to be delayed in mice treated with FLASH-PT compared to CONV-PT (Fig. 3A). This trend aligns with Sørensen et al. [36], who reported a clear sparing effect for FLASH-PT on skin tissue.

The regular MRI did not show any contrast-enhanced areas (Fig. 3B). With the current setup, we irradiated a brain sub-volume more than twice as large as that used by Suckert et al. [16], who reported that following 45 Gy CONV-PT, CELs became detectable on MRI after 10 weeks of follow-up. To account for the larger volume, we intentionally applied a lower dose to avoid distress to the mice; however, the dose of 22.5 Gy was insufficient to induce detectable CELs. To the best of our knowledge, in vivo FLASH preclinical studies investigating CELs are lacking. Our findings suggest that—independently on the irradiated brain volume—doses in the range used by Suckert et al. [16] may be required for CELs to be detectable on MRI.

Microglia activation at six-month post irradiation constitutes a sign of chronic neuroinflammation that can be a primary driver of neuronal damage [37]. Depending on the affected brain region, persistent neuroinflammation can contribute to different CNS pathologies. Identifying brain regions prone to sustained neuroinflammation could help improve post-treatment quality of life in tumour patients [38]. As shown in Fig. 4, for both FLASH-PT and CONV-PT, we found scattered activation and increased density of highly activated microglia along the lateral periventricular region. This pattern is consistent with the findings of Nexhipi et al. [17] and supports the higher sensitivity of this region, also seen in clinical data [39]. These results for both FLASH-PT and CONV-PT suggest that sub-structures within the periventricular area may be more radiosensitive than others, an important hypothesis for future studies. In addition, the microglia density maps (Fig. 4; Figs. S4.1 and S4.2 in the Supplementary Material) suggest differences in activation density between the midbrain- and forebrain-oriented periventricular regions. However, as Fig. 1A indicates that the periventricular region was not fully integrated in the irradiation field, this observation needs further validation. Notably, the observed pattern of increased activation density in the midbrain-oriented region is supported by the conclusions of Barko et al. [40], who reported transcriptomic signature differences between midbrain- and forebrain-resident microglia, suggesting that midbrain-microglia are more primed to engage in immune responses than forebrain-microglia. However, due to the relatively mild effects induced by the chosen dose, the range for detecting a potential reduction in toxicity under FLASH conditions was inherently limited and should be validated in future studies.

In conclusion, at 6 months after 22.5 Gy single fraction partial-brain FLASH-PT, no evidence of a FLASH sparing effect on microglia activation have been found. The analysis of skin reaction suggests a trend towards a protective FLASH effect, but under our treatment conditions, we could not demonstrate a neuroprotective effect. Nevertheless, we established a preclinical SOBP irradiation setup for sub-volume brain irradiation in mice that closely simulates clinical treatment. Future work will use this setup in a full-scale study to assess early skin reactions, MRI contrast enhancement, and long-term molecular markers. A future direction will be the establishment of dose–effect curves for microglia activation density after FLASH-PT, including more and earlier follow-up time points for all endpoints.

## Declaration of competing interest

The authors declare that they have no known competing financial interests or personal relationships that could have appeared to influence the work reported in this paper.

## Appendix A Supplementary data

Supplementary Data 1 Supplementary Material: Detailed description of the irradiation setup, dosimetry, proton transport simulations, staining protocol, analysed brain slices, and representative microglia activation maps.
