# Supplementary material for: Six-month evaluation of normal mouse brain side effects: Comparing FLASH and conventional proton partial brain irradiation
Source: Clin Transl Radiat Oncol. 2026 May 9;59:101183. doi: 10.1016/j.ctro.2026.101183 (PMC13195757; doi:10.1016/j.ctro.2026.101183)
Supplement: Supplementary Data 1 — Supplementary Material: Detailed description of the irradiation setup, dosimetry, proton transport simulations, staining protocol, analysed brain slices, and representative microglia activation maps. [file mmc1.docx]

**Supplementary material**

**Proton irradiation of mice**

Mouse irradiation was performed at the horizontal fixed-beam beamline in the experimental hall of OncoRay Dresden [1] adapting a previously described setup for partial mouse brain irradiation [2, 3]. The irradiations were accomplished with proton beams of 225 MeV energy using a dedicated 3D printed range modulator [4] to form a spread-out Bragg peak (SOBP) of 1.5 cm width in water. The SOBP was positioned in the mouse brain in its centre by a 24 cm thick polymethylmethacrylate (PMMA) range shifter in the beam path (Figure S1b). Two collimators shaped the lateral profile of the proton beam (Figure S2) before entering the mouse bedding unit [5]. The unit is positioned in lateral, vertical and horizontal direction by two motorized stages (LTM80F-100-HSM, LTM80P-75-HSM, OWIS GmbH, Staufen im Breisgau, Germany). All components of the setup and the respective distances are depicted in Figure S1 and beam parameters are summarized in Table 1. For the present mouse irradiation campaign, the workflow of previous campaigns [2] was adjusted taking into account the optimized proton radiography [3] and an altered positioning workflow, as described below.


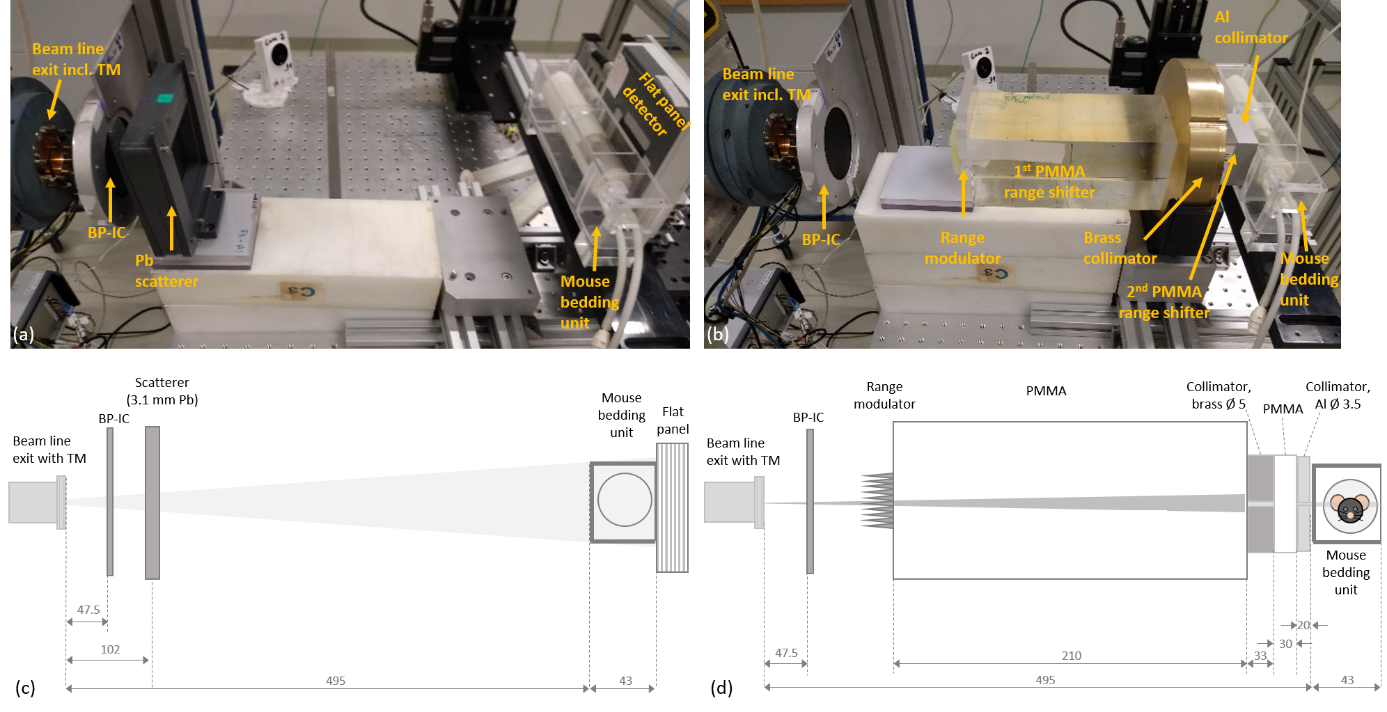


**Figure S1: Experimental setups used for proton radiography and proton treatment of the mice.** **(a)** and **(b)** photographs of the setup, whereas **(c)** and **(d)** are schematic representations comprising the individual components and respective distances. From left to right - the beam line exit, including transmission ionization chamber (TM), is followed by the Bragg peak ionization chamber (BP-IC) as a second independent beam monitor, the 3D-printed range modulator, then the PMMA range shifter and two collimators made of brass and aluminium with apertures of 5 mm and 3.5 mm openings, respectively, and end up with the mouse bedding unit enclosing the anaesthetized mouse. For proton radiography, a 3.1 mm lead scatterer replaces all components between BP-IC and mouse bedding unit and the flat panel detector was positioned as close as possible behind to the bedding unit. All measures are in mm.


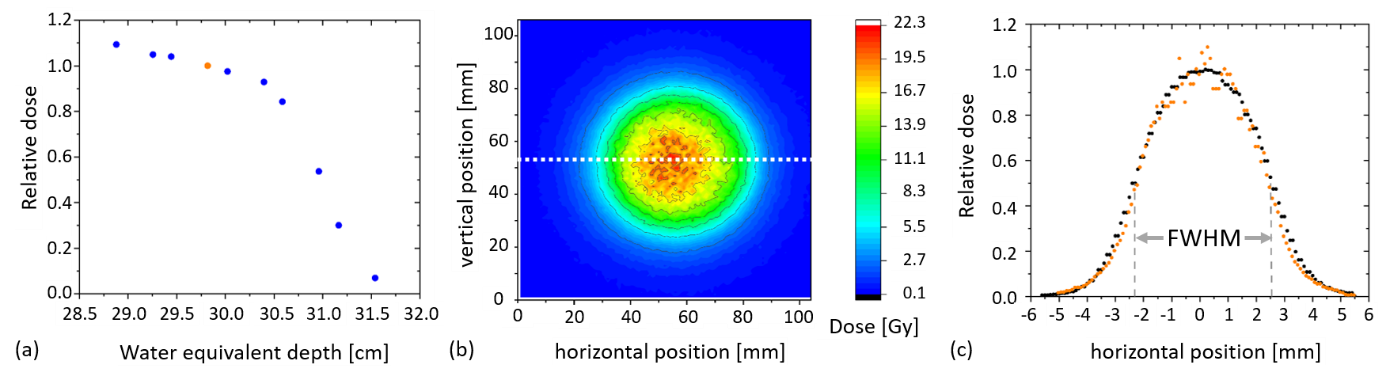


**Figure S2: Proton beam characteristics**. **(a)** Central depth dose profile of the modulated proton beam measured in the bedding unit at mouse head position by. The lateral profiles shown in (b,c) were recorded at the depth position marked in orange. **(b)** Colour coded lateral dose distribution derived from a Gafchromic EBT3 film irradiated in between two 5 mm PMMA slabs at mouse head position. **(c)** Lateral profiles extracted from the EBT3 film in (b) along the dotted line (orange) and measured with the Microdiamond detector (black dots) at the same depth. The profiles were normalized to the respective maxima.

**Proton radiography and mouse positioning**

Mouse positioning was performed deploying the optimized proton radiography imaging workflow described by Schneider et al. [3]. For image acquisition both, the range shifters and the collimators were removed and replaced by a lead scatterer closely behind the Bragg peak chamber (BP-IC) at beam line exit (Figure S1a and b) to have a broad field for imaging. A CMOS flat panel detector (C9320DK-02, Hamamatsu Photonics K.K., Hamamatsu City, Japan) at a fixed position acquired images of 52.8x52.8 mm^2^ size at a frame rate of 8.4 Hz. The software for detector read-out was custom written in-house using the National Instruments Software Interface NI-IMAQ (version 3.7, National Instruments Corporation, Austin, USA). Using 200 MeV proton beam energy (beam current 0.1 nA, irradiation time 1 s) an acceptable image quality was achieved (Figure S3, middle) although the distance between detector and scatterer was reduced by three-quarters compared to previous studies (0.45 m vs. 2 m). In this setting, an imaging dose of 50 mGy was delivered to each mouse as measured at mouse position with a Semiflex ionization chamber (2305, PTW Dosimetry, Freiburg, Germany), calibrated for dose absorbed to water by ^60^Co photons and corrected for temperature and air pressure, and applying radiation quality correction factor kq = 1.02 estimated from TRS-398 [6]. For positioning, the software RadiAiDD (https://github.com/jo-mueller/RadiAiDD, Version 0.1.0) was applied calculating the correction vectors for the motorized stages based on radiography images of collimator and mouse [3]. In a first step, beam (isocenter) position was defined by taking a proton radiography of the collimator (225 MeV, 0.1 nA, 150 ms). Second, the proton radiography of the actual mouse was co-registered to a high-resolution proton radiography previously acquired of an age-matched C57BL/6 mouse (Figure S3, left). For this purpose, the target volume (cross), beam isocenter (circle) and landmarks for positioning (coloured dots) were marked in the high-resolution image (Figure S3, left). The same landmarks were set in the actual mouse radiography (Figure S3, middle) to facilitate co-registration of both images. The success of the co-registration could be followed by the overlay image on the right side of Figure S3. Finally, RadiAiDD calculated the new stage coordinates to move the mouse in treatment position, i.e. position the target volume (hippocampal area) in front of the aperture of the last collimator.

**Dosimetry**

Lateral and depth dose profiles of the proton field were measured with a microdiamond detector (T31022-152249, PTW Dosimetry, kq = 1.022 [7]), readout by Unidos electrometer (10001, PTW Dosimetry) at mouse head position, i.e. behind 7.3 mm thick PMMA representing the entrance wall of the bedding unit (2.3 mm PMMA) and one-half of the mouse brain (∼6 mm water equivalent thickness). For proton dosimetry the microdiamond detector was cross-calibrated against an Advanced Markus chamber (34045, PTW Dosimetry), readout by Tango electrometer (10052, PTW Dosimetry). Cross-calibration was done by irradiating both detectors at same position in a water phantom using a large homogenous proton field of 150 MeV energy. The strong collimation caused a slope in the SOBP (Figure S2a) resulting in ± 6 % dose inhomogeneity over the mouse head assuming a thickness of 12 mm for the mouse head. Lateral profiles (Figure S2c) were recorded at central position (Figure S2a, orange spot) with the microdiamond on linear stages and by Gafchromic EBT3 films (LOT 11192002, Ashland Advanced Materials, Bridgewater NJ, USA) in between two 5 mm thick PMMA slabs. The EBT3 films (example in Figure S2b) were scanned with a flatbed scanner (11000 XL, EPSON Expression) translating the pixel values to dose by means of a previously obtained calibration in a large homogenous proton field of 150 MeV energy. The full-width at half-maximum of the beam has a diameter of about 5 mm; the dose inhomogeneity in the central part of the beam spot was approximately ± 5 %. In order to fulfil the UHDR requirement a dose rate of 268 Gy/s at central axis was applied (Table 1). The dose delivery at conventional dose rate was monitored by means of the transmission ionisation chamber (TM34058, PTW Dosimetry) integrated in and the Bragg Peak chamber (BP-IC) (34070-2.5, PTW Dosimetry), operated at a chamber voltage of 400 V and positioned close to the beam line exit (Figure S1c and d). Both chambers were cross-calibrated on a daily basis against the microdiamond at mouse head position. The TM provided monitor units for controlled beam delivery at conventional dose rate, but the high recombination effects in this chamber prevent its application at UHDR. The BP-IC was readout with a Keithley electrometer (6514; Keithley Instruments, Ohio, USA), whereas the reading was corrected for recombination effects at UHDR using a correction factor of kS = 1.04 determined by a Jaffé diagram. Prior to mouse irradiation, the proton pulse length for UHDR treatment was determined by BP-IC measurements taken into account the cross-calibration at conventional dose rate and the recombination correction. Equivalence of dose delivery at both regimes was verified by EBT3 film measurements in between two 5 mm thick PMMA slabs at mouse head position and by microdiamond.


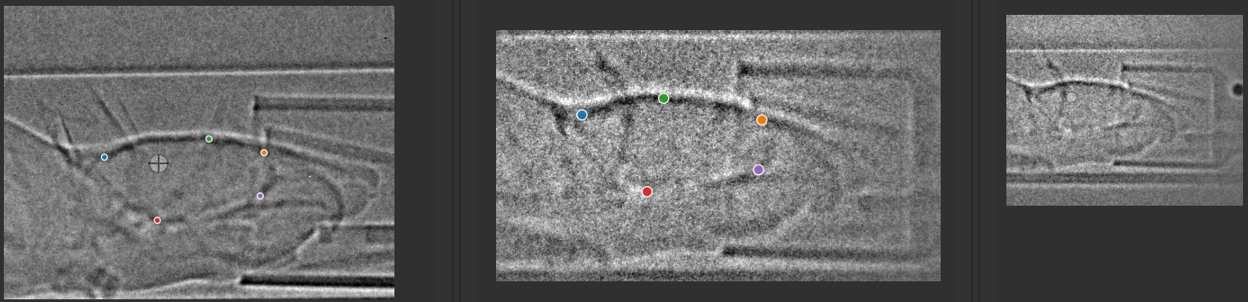


**Figure S3: Proton radiography based positioning workflow**. Matching of the proton radiography of the actual mouse (middle) to the high-resolution radiography of an age-matched C57BL/6 mouse (left) including the positions of collimator (circle) and target (cross) using the RadiAiDD software. The right image proof the success of co-registration by a sliding window before the software calculates the new target coordinates for the motorized stages.

**Dose simulations**

Monte Carlo (MC) simulations to obtain the dose distributions for two mice (M02 and M03) were performed with TOPAS (Tool for Particle Simulation) version 3.9 [8, 9] similar to what was described earlier [2]. However, here the proton source was placed directly upstream to the last collimator and implemented as a flat circular positional distribution (diameter = 5 mm) without angular distribution. The proton energy distribution behind the range modulator was sampled from an energy spectrum that had been previously simulated and scored with another MC code, FLUKA, by implementing the beamline design in FLUKA, considering the exact modulator geometry [4]. The cone-beam computed tomography (CBCT) images (SAIGRT [10]) of the mice in the bedding unit were imported into TOPAS using the Schneider material conversion method to convert CBCT numbers (image intensities) voxel wise into predefined materials [11]. Simulations were performed with 10^9^ primary protons per mouse on the Linux high performance cluster LiDO3 at TU Dortmund University. The relative mean statistical uncertainty of voxels with dose values above 1% of the dose maximum was below 1%.

**Mice list with relative microglia density values and density maps:**

**Table S1:** List of mice that have been employed in the study. M07, M11, M12 brains were not sliced and not analysed. The density of microglia activation is expressed as cells/mm^2^. The whole-brain area was considered for this analysis.

| **ID** | **Treatment group** | **Slice N°** | **Total microglia density (cells/mm^2^)** | **Non-activated density (cells/mm^2^)** | **Activated density (cells/mm^2^)** | **Highly activated density (cells/mm^2^)** |
| --- | --- | --- | --- | --- | --- | --- |
| M01 | FLASH-PT | 5 | 45,420 | 27,144 | 16,184 | 2,091 |
| M02 | FLASH-PT | 6 | 80,883 | 31,549 | 41,754 | 7,579 |
| M03 | CONV-PT | 6 | 96,764 | 38,044 | 49,069 | 9,651 |
| M04 | FLASH-PT | 5 | 58,210 | 39,060 | 16,826 | 2,323 |
| M05 | CONV-PT | 6 | 35,433 | 23,386 | 10,735 | 1,311 |
| M06 | CONV-PT | 6 | 44,646 | 29,767 | 13,109 | 1,769 |
| M07 | CONTROL | - | - | - | - | - |
| M08 | FLASH-PT | 5 | 46,254 | 32,755 | 12,329 | 1,169 |
| M09 | CONV-PT | 5 | 45,694 | 27,980 | 15,460 | 2,253 |
| M10 | CONV-PT | 5 | 52,765 | 40,787 | 10,714 | 1,263 |
| M11 | CONV-PT | - | - | - | - | - |
| M12 | FLASH-PT | - | - | - | - | - |
| M13 | FLASH-PT | 6 | 42,007 | 29,367 | 11,371 | 1,268 |
| M14 | CONTROL | 5 | 45,362 | 34,706 | 9,934 | 0,722 |

**
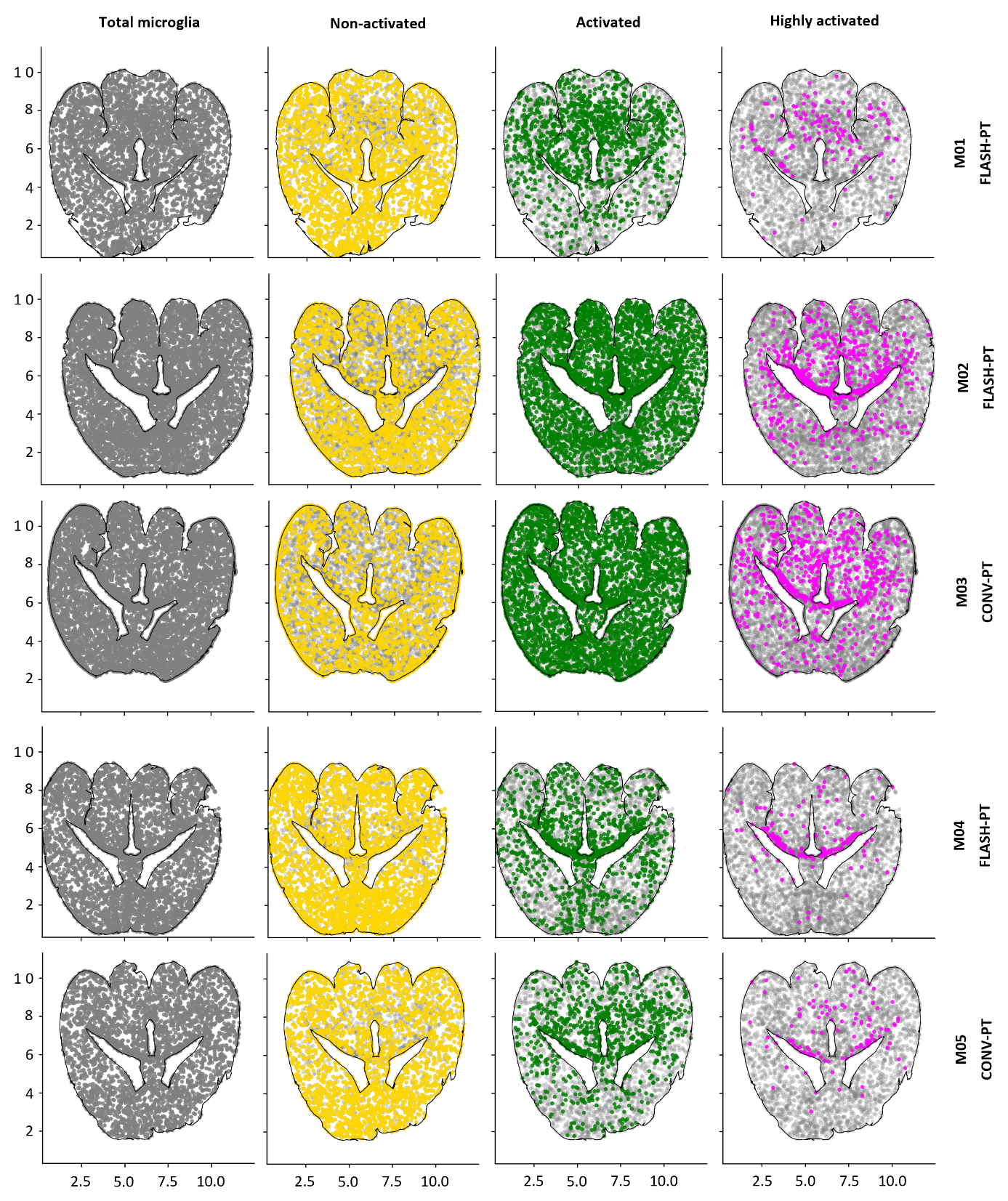
**

**Figure S4.1: Microglia activation density maps for mice from M01 to M05**. The figure represents the microglia distribution for mice in different treatment groups at matched irradiation depths. The images were created by plotting the centre-of-mass coordinate point of each microglia in the slice and then color-coded based on the activation status. Total microglia population is reported as reference in grey colour in all the maps. Scale system is in mm.

**
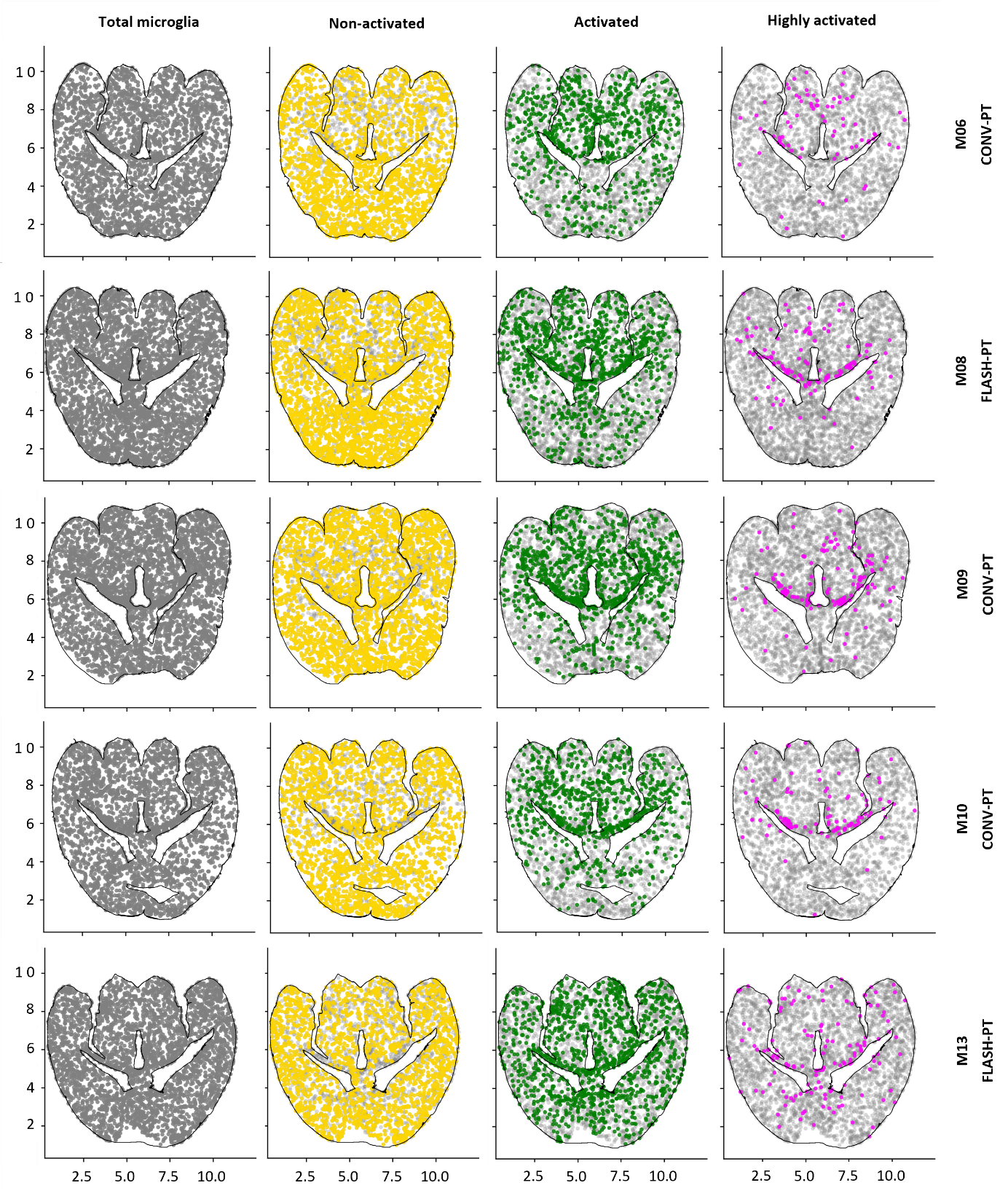
**

**Figure S4.2: Microglia activation density maps for mice from M06 to M13**. The figure represents the microglia distribution for mice in different treatment groups at matched irradiation depths. The images were created by plotting the centre-of-mass coordinate point of each microglia in the slice and then color-coded based on the activation status. Total microglia population is reported as reference in grey colour in all the maps. Scale system is in mm.

**Antibody list with histology protocol**

**Table S2:** Blocking buffer and antibody diluent composition.

| **Solution** | **Components** |
| --- | --- |
| Blocking buffer (A) | 1X Roti-Block 0.1% Triton X-100 Distilled water |
| Antibody diluent (B) | 1X Roti-Block Distilled water |

**Table S3:** Primary and secondary antibodies used for immunofluorescence.

| **Target** | **Company** | **Catalog No.** | **Host** | **Dilution** | **Secondary Antibody (Dilution)** |
| --- | --- | --- | --- | --- | --- |
| Iba1 | Novus Biological | NB100-1028 | Goat | 1:500 | Donkey anti-goat AlexaFluor594, Invitrogen A11058 (1:500) |

**Table S4:** Other reagents and buffer preparations.

| **Reagent** | **Details** |
| --- | --- |
| Citrate buffer pH 6 | Solution A: 0.1 M Citric Acid (14.4 ml / 800 ml) Solution B: 0.1 M Sodium Citrate (65.6 ml / 800 ml) |
| Roti®-Block | Roth A151.1 (1:10 in distilled water) |
| DAPI | Abcam ab228549 (1:1000 in PBS) |

**Table S5:** Immunofluorescence staining protocol.

| **Step** | **Details** |
| --- | --- |
| Dewaxing | 3 × 8 min in Xylene under the hood |
| Rehydration | 1 min each in decreasing ethanol series |
| PBS rinse | 2 min |
| Antigen retrieval | 26 min at 630 W in citrate buffer (pH 6) using microwave |
| Cooling | 15 min on ice |
| PBS rinse | 2 min |
| Hydrophobic barrier | Draw with Pap-Pen; avoid drying out |
| Blocking | 30 min at RT in blocking buffer (A), no wash |
| Primary antibody | 60 min at RT in humid chamber, diluted in (B) |
| PBS wash | 1× rinse, then 2× 5 min washes |
| *(From here: protect from light)* |  |
| Secondary antibody | 60 min at RT in humid chamber, diluted in (B) |
| PBS wash | 1× rinse, then 2× 5 min washes |
| DAPI staining | 10 min at RT in 1:1000 DAPI in PBS |
| PBS wash | 1× rinse, then 2× 5 min washes |
| Mounting | Coverslip with fluorescence mounting medium |
| Drying | Overnight in the dark at RT |
| Storage | 4°C |

**References**

[1] Horst F, Bodenstein E, Baumann M, Beyreuther E, Bokor J, Enghardt W, et al. The research beamlines at the Dresden proton therapy facility: available infrastructure and experimental capabilities. Front Oncol [Internet]. 2025 June 5;15. Available from: <http://dx.doi.org/10.3389/fonc.2025.1594973>

[2] Suckert T, Müller J, Beyreuther E, Azadegan B, Brüggemann A, Bütof R, et al. High-precision image-guided proton irradiation of mouse brain sub-volumes. Radiotherapy and Oncology [Internet]. 2020 May;146:205–12. Available from: <http://dx.doi.org/10.1016/j.radonc.2020.02.023>

[3] Schneider M, Bodenstein E, Bock J, Dietrich A, Gantz S, Heuchel L, et al. Combined proton radiography and irradiation for high-precision preclinical studies in small animals. Front Oncol [Internet]. 2022 Aug 31;12. Available from: <http://dx.doi.org/10.3389/fonc.2022.982417>

[4] Horst F, Beyreuther E, Bodenstein E, Gantz S, Misseroni D, Pugno NM, et al. Passive SOBP generation from a static proton pencil beam using 3D-printed range modulators for FLASH experiments. Front Phys [Internet]. 2023 July 4;11. Available from: <http://dx.doi.org/10.3389/fphy.2023.1213779>

[5] Müller J, Schürer M, Neubert C, Tillner F, Beyreuther E, Suckert T, et al. Multi-modality bedding platform for combined imaging and irradiation of mice. Biomed Phys Eng Express [Internet]. 2020 Apr 14;6(3):037003. Available from: <http://dx.doi.org/10.1088/2057-1976/ab79f1>

[6] Absorbed Dose Determination in External Beam Radiotherapy [Internet]. Technical Reports Series. INTERNATIONAL ATOMIC ENERGY AGENCY; 2024. Available from: <http://dx.doi.org/10.61092/iaea.ve7q-y94k>

[7] Sotiropoulos M, Prezado Y. Radiation quality correction factors for improved dosimetry in preclinical minibeam radiotherapy. Medical Physics [Internet]. 2022 Aug 18;49(10):6716–27. Available from: <http://dx.doi.org/10.1002/mp.15838>

[8] Perl J, Shin J, Schümann J, Faddegon B, Paganetti H. TOPAS: An innovative proton Monte Carlo platform for research and clinical applications. Medical Physics [Internet]. 2012 Oct 19;39(11):6818–37. Available from: <http://dx.doi.org/10.1118/1.4758060>

[9] Faddegon B, Ramos-Méndez J, Schuemann J, McNamara A, Shin J, Perl J, et al. The TOPAS tool for particle simulation, a Monte Carlo simulation tool for physics, biology and clinical research. Physica Medica [Internet]. 2020 Apr;72:114–21. Available from: <http://dx.doi.org/10.1016/j.ejmp.2020.03.019>

[10] Tillner F, Thute P, Löck S, Dietrich A, Fursov A, Haase R et al. Precise image-guided irradiation of small animals: a flexible non-profit platform. Physics in Medicine & Biology 61.8 (2016): 3084-3108.

[11] Schneider W, Bortfeld T, Schlegel W. Correlation between CT numbers and tissue parameters needed for Monte Carlo simulations of clinical dose distributions. Phys Med Biol [Internet]. 2000 Jan 25;45(2):459–78. Available from: <http://dx.doi.org/10.1088/0031-9155/45/2/314>
